# Discovery of Prognostic Signature Genes for Overall Survival Prediction in Gastric Cancer

**DOI:** 10.1155/2020/5479279

**Published:** 2020-08-25

**Authors:** Changyuan Meng, Shusen Xia, Yi He, Xiaolong Tang, Guangjun Zhang, Tong Zhou

**Affiliations:** ^1^The Second Department of Gastrointestinal Surgery, The Affiliated Hospital of North Sichuan Medical College, Nanchong, Sichuan, China; ^2^Institute of Hepatobiliary, Pancreatic and Intestinal Disease, North Sichuan Medical College, Nanchong, Sichuan, China

## Abstract

**Background:**

Gastric cancer (GC) is one of the most common malignant tumors in the digestive system with high mortality globally. However, the biomarkers that accurately predict the prognosis are still lacking. Therefore, it is important to screen for novel prognostic markers and therapeutic targets.

**Methods:**

We conducted differential expression analysis and survival analysis to screen out the prognostic genes. A stepwise method was employed to select a subset of genes in the multivariable Cox model. Overrepresentation enrichment analysis (ORA) was used to search for the pathways associated with poor prognosis.

**Results:**

In this study, we designed a seven-gene-signature-based Cox model to stratify the GC samples into high-risk and low-risk groups. The survival analysis revealed that the high-risk and low-risk groups exhibited significantly different prognostic outcomes in both the training and validation datasets. Specifically, *CGB5*, *IGFBP1*, *OLFML2B*, *RAI14*, *SERPINE1*, *IQSEC2*, and *MPND* were selected by the multivariable Cox model. Functionally, PI3K-Akt signaling pathway and platelet-derived growth factor receptor (PDGFR) were found to be hyperactive in the high-risk group. The multivariable Cox regression analysis revealed that the risk stratification based on the seven-gene-signature-based Cox model was independent of other prognostic factors such as TNM stages, age, and gender.

**Conclusion:**

In conclusion, we aimed at developing a model to predict the prognosis of gastric cancer. The predictive model could not only effectively predict the risk of GC but also be beneficial to the development of therapeutic strategies.

## 1. Introduction

Gastric cancer (GC) is the fifth most common malignancies worldwide in 2018, accounting for 5.7% of total new cases and 8.2% of cancer-related deaths [1]. Most GC cases are from developing countries, and increased prevalence in the younger population is observed [2]. The major risk factor for GC is Helicobacter pylori infection, and its eradication is considered as the most critical for the prevention of GC [3]. Meanwhile, GC often exhibits a high metastasis rate, and most GC patients are not effectively diagnosed at early stages, where surgical resection could become unavailable, which leads to the generally poor prognoses of GC patients [4]. Therefore, there is an urgent need to focus on accurately identifying markers of prognostic value, in order to provide personalized treatment strategies and to improve the survival of GC patients.

Thanks to the development in sequencing technologies, the utilization of gene expression data makes it possible to explore the molecular background of GC. GC is considered a heterogeneous disease, and so far, several classifications of molecular subtypes of GC have been established. The genomic studies reveal that mutations in *CDH1*, *ERBB4*, *MET*, and *CD44* are closely associated with poor prognosis in gastric cancer [5, 6]. A recent research has reported 4 molecular subtypes that can be identified using immunohistochemical analysis, the Pentaplex assay and certain gene expression (*VIM*, *ZEB1*, *MDM2*, and *CDKN1A*), which are the mesenchymal-like type, Microsatellite-unstable type, tumor protein 53- (TP53-) active and TP53-inactive types, each of them characterized by distinctive prognosis and recurrence patterns [7]. A 19-gene signature was developed to distinguish grades and stages of GC, with an overall accuracy at 79.6%, but among those detected genes, only *CLDN7*, *CLDN1*, and *DPT* exhibited significantly varied expression when compared with normal tissues [8]. Notably, another study has presented a prognostic scoring system developed with 53 gene signatures for GC, including well-reported cancer hallmark genes like *FGFR4*, *CEP55*, and *MCM2* [9]. However, the identification of biomarkers with high prognostic efficacy and the establishment of prognostic scoring with fewer but more effective markers are still essential. In the present study, we aimed at identifying a combination of prognostic genes to predict the risk of GC and stratify the samples, which might be beneficial to the development of therapeutic strategies.

## 2. Materials and Methods

### 2.1. Data Acquisition

The gene expression data from the Cancer Genome Atlas (TCGA) project [10] were collected from the UCSC Xena database [11]. We only retained 350 gastric cancer and 32 normal tissues with detailed clinical information. The independent validation dataset was collected from Gene Expression Omnibus [12] (GEO) with accession GSE84433. The TCGA dataset was normalized by log-transforming the FPKM (Fragment Per Kilobase Per Million Reads) +1. The microarray gene expression data of GSE84433 was normalized following a previous study [13]. The former dataset was used for selecting genes for model training, and the latter was used to validate the model performance.

### 2.2. Selection of Prognostic Genes in Gastric Cancer

To select the prognostic genes in gastric cancer, we first conducted differential expression analysis between the gastric cancer and adjacent normal tissues. Wilcoxon rank -sum test and fold change were employed to identify the upregulated and downregulated genes in gastric cancer. The adjusted *p* value of 0.05 and fold change of 2 were chosen as the thresholds for the differentially expressed genes (DEGs). Furthermore, a univariate Cox regression analysis was conducted to identify those overall survival-associated genes from the DEGs (*p* < 0.05). The optimal combination of prognostic genes was selected by a stepwise method with the R language *step* function. The gene sets with minimal Akaike information criterion (AIC) values were selected as the predictors in the multivariable Cox model.

### 2.3. Overrepresentation Enrichment Analysis (ORA)

The ORA was employed to identify the pathways enriched by a given gene set. The Fisher's exact test was used to test the statistical significance of each pathway. The analysis and visualization was implemented in the R package *clusterProfiler* [14].

### 2.4. Discovery of Drug-Target

The upregulated genes in the gastric cancer samples with worse prognosis were used to identify the potential therapeutic targets. The drug-target data was curated by R *maftools* package [15] *drugInteractions*, which searched for the drugs based on the genes.

### 2.5. Survival Analysis

The Cox proportional hazard regression analysis was employed to identify genes associated with the overall survival of gastric cancer. The genes were binarized based on the median of expression levels. The samples were stratified into high-risk and low-risk groups based on the median of risk scores estimated by the Cox model.

## 3. Results

### 3.1. Identification of Prognostic Genes in Gastric Cancer

**Figure 1:**
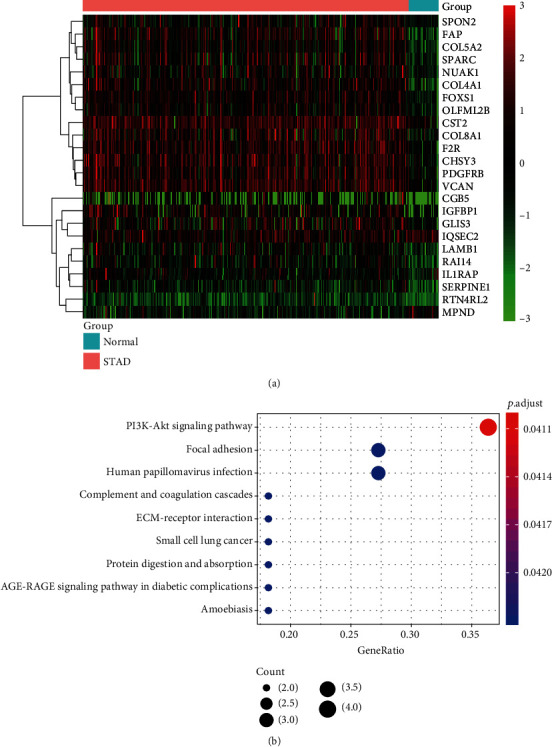
The expression patterns and functionalities of prognostic genes in GC. (a) The expression patterns of the 24 prognostic genes selected by differential expression analysis and univariable Cox regression analysis. The expression levels were scaled to -3 to 3. (b) The pathways enriched by the 24 prognostic genes. The node color and size represent the statistical significance and the number of genes included in the pathway.

To identify the prognostic genes in gastric cancer, we first collected gene expression data of 350 gastric cancer and 32 normal tissues from the Cancer Genome Atlas (TCGA) project. Subsequently, we conducted a differential expression analysis of the gene expression data by comparing the tumor with the normal tissues. Moreover, we also conducted Cox regression analysis to identify the upregulated and downregulated genes that were associated with overall survival (OS) of the gastric cancer (adjusted *p* value < 0.05 and fold change > 1). Specifically, we identified a total of 24 prognostic genes in gastric cancer including 22 upregulated and 2 downregulated genes (Supplementary Table S1, Figure 1(a), adjusted *p* value < 0.05). To reveal the functionality of these genes, we conducted overrepresentation enrichment analysis (ORA) of the 24 prognostic genes and found that these genes were enriched in cancer-related pathways, such as PI3K-Akt signaling pathway, focal adhesion, complement and coagulation cascades, and ECM-receptor interaction. These results indicated that these prognostic genes could not only act as predictors for OS prediction but also be used for interpreting the reason of the worse prognosis in gastric cancer.

### 3.2. Construction and *In Silico* Validation of Multivariable Cox Model for OS Prediction

**Figure 2:**
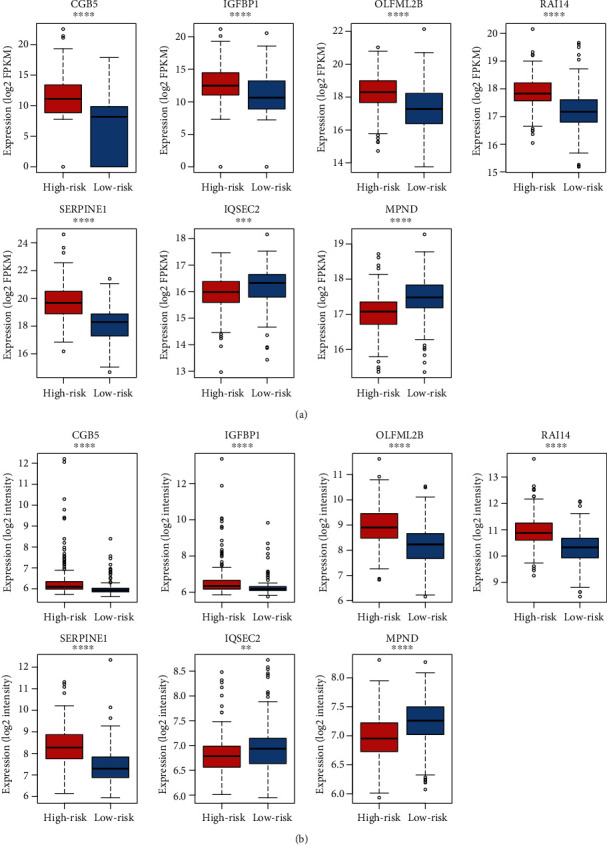
The gene expression levels of the seven gene signatures in the two risk groups. The differential expression levels of the seven prognostic genes between the high-risk and low-risk groups in TCGA (a) and GSE84433 (b) datasets, which were referred to as training and validation datasets, respectively. The red and blue boxes represent the high-risk and low-risk groups. (^∗^ < 0.05, ^∗∗^ < 0.01, ^∗∗∗^ < 0.001, and ^∗∗∗∗^ < 0.0001).

**Figure 3:**
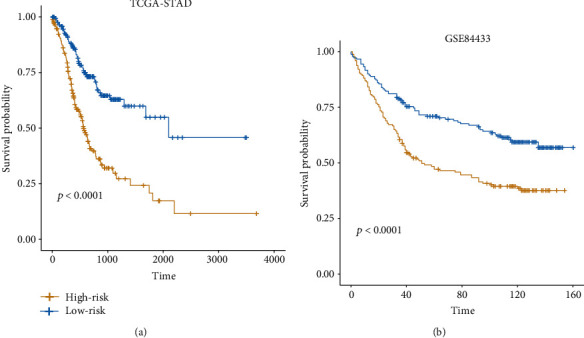
The Kaplan-Meier (KM) curves of the two risk groups in the training and validation datasets. The difference of the probabilities of the overall survival in the training (a) and validation (b) datasets. The log-rank test was used to test the differences between the high-risk and low-risk groups. The yellow and blue lines represent the high-risk and low-risk groups.

With the 24 prognostic genes, a stepwise method was employed to identify a subset of genes in the multivariate analysis. Specifically, *CGB5*, *IGFBP1*, *OLFML2B*, *RAI14*, *SERPINE1*, *IQSEC2*, and *MPND* were selected by the multivariable Cox model (Table 1). The samples in TCGA and the validation cohorts were then stratified into high-risk and low-risk groups by the median of the risk scores. The seven signature genes were observed to be remarkably differentially expressed between the two groups in both TCGA (Figure 2(a)) and the validation cohorts (Figure 2(b)). The log-rank test revealed that the high-risk group had a significantly worse prognosis than the low-risk group (Figure 3(a)). Moreover, the two groups in the validation cohort were also observed to have significantly different prognostic outcomes in the independent dataset (Figure 3(b)). Furthermore, we compared the seven-gene-signature with others by Cui et al. [8] and Wang et al. [9], and our proposed gene signatures exhibited higher performance than the others (Supplementary Table S2). These results suggested that the seven-gene-signature-based Cox model was capable of predicting the overall survival of gastric cancer.

### 3.3. The Risk Stratification Is an Independent Prognostic Factor in Gastric Cancer

**Figure 4:**
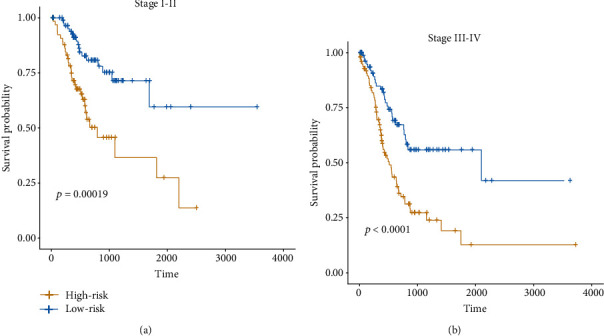
The differential prognostic outcomes in the early-stage and advanced GC. The early-stage and advanced GC were defined by those samples with TNM stage I-II, and III-IV, respectively. The KM curves of the early-stage and advanced GC were displayed in (a) and (b). Log-rank test was used to test the difference.

To demonstrate the independence of the risk stratification, we built a multivariable Cox model on the risk stratification with TNM stage, age, and gender as cofactors. Consistently, the risk stratification still maintained higher statistical significance than the TNM stage in the multivariable Cox model (Table 2). Moreover, the older age was an unfavorable factor in gastric cancer. Consistently, we found that high-risk group had a shorter overall survival than the low-risk group in both samples with early stage (I-II) and those with advanced stage (III-IV) (Figures 4(a) and 4(b)). These results indicated that the risk stratification is an independent prognostic factor in gastric cancer.

### 3.4. The Biomarkers and Pathways Associated with OS in Gastric Cancer

**Figure 5:**
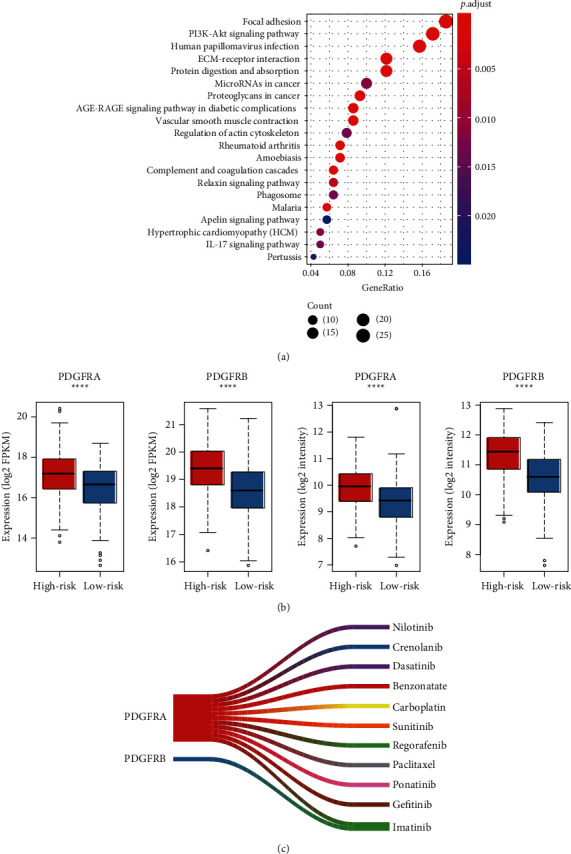
The critical biomarkers and pathways in the high-risk group of GC. (a) The pathways enriched by the upregulated genes in the high-risk group of GC. (b) The differential expression levels of *PDGFRA* and *PDGFRB* between the high-risk and low-risk groups. The left two panels represent the data in the TCGA cohort, and the right two represent the GSE84433 cohort. (c) The drugs that potentially inhibit the PDGFRA or PDGFRB. (^∗^ < 0.05, ^∗∗^ < 0.01, ^∗∗∗^ < 0.001, and ^∗∗∗∗^ < 0.0001).

To further interpret the underlying mechanism and key molecules resulting in poor outcome in gastric cancer, we compared the gene expression profiles of the high-risk group with those of the low-risk group. ORA analysis of these upregulated genes in high-risk group revealed that PI3K-Akt signaling pathway and tumor microenvironment-related pathways such as focal adhesion, ECM-receptor interaction, and complement and coagulation cascades might play key roles in the high-risk group of gastric cancer (Figure 5(a)). Notably, two receptors of growth factor in PI3K-Akt signaling, PDGFRA and PDGFRB, were significantly upregulated in the high-risk group of both TCGA and validation cohorts (Figure 5(b)). Moreover, drugs including Nilotinib, Crenolanib, Dasatinib, Benzonatate, Carboplatin, Sunitinib, Regorafenib, Paclitaxel, Ponatinib, Gefitinib, and Imatinib were found to target the two receptors, suggesting that the high-risk group might be treated by these PDGFR inhibitors.

## 4. Discussion

Gastric cancer (GC) is one of the most common malignant tumors in the digestive system. Here, we designed a seven-gene-signature-based Cox model to stratify the GC samples into high-risk and low-risk groups. The survival analysis revealed that the high-risk and low-risk groups exhibited significantly different prognostic outcomes in both the training and validation datasets, suggesting that the seven-gene-signature-based Cox model was capable of predicting the overall survival of gastric cancer.

Specifically, *CGB5*, *IGFBP1*, *OLFML2B*, *RAI14*, *SERPINE1*, *IQSEC2*, and *MPND* were selected by the multivariable Cox model. CGB5 is one of the key hCG*β* encoding genes, which acts as a proangiogenic factor in some tumors [16, 17], suggesting that CGB5 might also promote angiogenesis in gastric cancer. IGFBP1 is involved in the insulin signaling pathway [18], which also participates in the regulation of the PI3K-Akt signaling pathway [19–21]. In accordance with this, the PI3K-Akt signaling pathway was found to be hyperactive in the high-risk group. Notably, the platelet-derived growth factor receptor [22, 23], *PDGFRA* and *PDGFRB*, was significantly upregulated in the high-risk group, further demonstrating that the PDGF/PDGFR and PI3K-Akt signaling pathway were responsible for the worse prognostic outcome and might be the potential therapeutic targets in gastric cancer. Among the drugs inhibiting the activity of PDGFR, Crenolanib [24] and Regorafenib [25] have been found to act as potential targeted therapies in gastric cancer. The remaining prognostic genes such as *OLFML2B*, *RAI14*, *SERPINE1*, and *MPND* were also reported to be dysregulated and associated with poor prognosis in gastric cancer [26–29].

The further evaluation of the risk stratification revealed that it is an independent prognostic factor in gastric cancer. With the TNM stage, age, and gender as cofactors, the risk stratification still maintained statistical significance in the multivariable Cox model, indicating that the risk stratification, combined with TNM stage, age, and gender, had the potential to be applied in OS prediction of gastric cancer.

In summary, we aimed at developing a combination of prognostic gene signatures and building a robust model for GC risk prediction. The predictive model could not only effectively predict the risk of GC but also be beneficial to the development of therapeutic strategies.

## Figures and Tables

**Table 1:** The hazard ratio and statistical significance of the seven signature genes in univariate and multivariate analyses.

Genes	Univariate analysis	*p* value	Multivariate analysis	*p* value
	HR (95% CI)		HR (95% CI)	
*CGB5*	1.79 (1.28-2.50)	6.02E-04	1.78 (1.27-2.50)	8.99E-04
*IGFBP1*	1.66 (1.19-2.30)	2.63E-03	1.33 (0.95-1.87)	1.02E-01
*OLFML2B*	1.77 (1.27-2.48)	7.60E-04	1.64 (1.13-2.38)	9.36E-03
*RAI14*	1.82 (1.30-2.54)	4.19E-04	1.35 (0.93-1.96)	1.16E-01
*SERPINE1*	1.95 (1.39-2.72)	9.32E-05	1.62 (1.14-2.30)	6.66E-03
*IQSEC2*	0.71 (0.51-0.99)	4.35E-02	0.77 (0.55-1.07)	1.24E-01
*MPND*	0.65 (0.47-0.91)	1.14E-02	0.73 (0.51-1.03)	7.67E-02

**Table 2:** The multivariate Cox analysis of the risk stratification, TNM stage, age, and gender.

Factors	HR (95% CI)	*p* value
Risk stratification		
High-risk	1 (reference)	
Low-risk	0.40 (0.28-0.58)	1.41E-06
TNM stage		
I	1 (reference)	
II	1.44 (0.75-2.80)	2.73E-01
III	1.99 (1.07-3.69)	3.02E-02
IV	3.82 (1.86-7.84)	2.58E-04
Age	1.03 (1.01-1.04)	5.81E-03
Gender		
Female	1 (reference)	
Male	1.04 (0.72-1.51)	8.31E-01

## Data Availability

TCGA data were collected from UCSC Xena database and the independent validation dataset was collected from Gene Expression Omnibus with accession GSE84433.

## Supplementary Materials

Supplementary Materials Description of the two supplementary tables. Supplementary Table 1: 22 upregulated and 2 downregulated genes in gastric cancer. Supplementary Table 2: other studies involved in our proposed signatures.

## References

[1] Bray F., Ferlay J., Soerjomataram I., Siegel R. L., Torre L. A., Jemal A. (2018). Global cancer statistics 2018: GLOBOCAN estimates of incidence and mortality worldwide for 36 cancers in 185 countries. *CA: A Cancer Journal for Clinicians*.

[2] den Hoed C. M., Kuipers E. J. (2016). Gastric cancer: how can we reduce the incidence of this disease?. *Current Gastroenterology Reports*.

[3] Eusebi L. H., Telese A., Marasco G., Bazzoli F., Zagari R. M. (2020). Gastric cancer prevention strategies: a global perspective. *Journal of Gastroenterology and Hepatology*.

[4] Song Z., Wu Y., Yang J., Yang D., Fang X. (2017). Progress in the treatment of advanced gastric cancer. *Tumour Biology*.

[5] Corso G., Carvalho J., Marrelli D. (2013). Somatic mutations and deletions of the E-cadherin gene predict poor survival of patients with gastric cancer. *Journal of Clinical Oncology*.

[6] Shi J., Yao D., Liu W. (2012). Frequent gene amplification predicts poor prognosis in gastric cancer. *International Journal of Molecular Sciences*.

[7] Cristescu R., Lee J., Nebozhyn M. (2015). Molecular analysis of gastric cancer identifies subtypes associated with distinct clinical outcomes. *Nature Medicine*.

[8] Cui J., Li F., Wang G., Fang X., Puett J. D., Xu Y. (2011). Gene-expression signatures can distinguish gastric cancer grades and stages. *PLoS One*.

[9] Wang P., Wang Y., Hang B., Zou X., Mao J. H. (2016). A novel gene expression-based prognostic scoring system to predict survival in gastric cancer. *Oncotarget*.

[10] The Cancer Genome Atlas Research Network (2014). Comprehensive molecular characterization of gastric adenocarcinoma. *Nature*.

[11] Goldman M., Craft B., Hastie M. (2018). The UCSC Xena Platform for cancer genomics data visualization and interpretation.

[12] Barrett T., Wilhite S. E., Ledoux P. (2013). NCBI GEO: archive for functional genomics data sets--update. *Nucleic Acids Research*.

[13] Yoon S. J., Park J., Shin Y. (2020). Deconvolution of diffuse gastric cancer and the suppression of CD34 on the BALB/c nude mice model. *BMC Cancer*.

[14] Yu G., Wang L. G., Han Y., He Q. Y. (2012). clusterProfiler: an R package for comparing biological themes among gene clusters. *OMICS: A Journal of Integrative Biology*.

[15] Mayakonda A., Lin D. C., Assenov Y., Plass C., Koeffler H. P. (2018). Maftools: efficient and comprehensive analysis of somatic variants in cancer. *Genome Research*.

[16] Schanz A., Lukosz M., Hess A. P., Baston-Bust D. M., Krussel J. S., Heiss C. (2015). hCG stimulates angiogenic signals in lymphatic endothelial and circulating angiogenic cells. *Journal of Reproductive Immunology*.

[17] Brouillet S., Hoffmann P., Chauvet S. (2012). Revisiting the role of hCG: new regulation of the angiogenic factor EG-VEGF and its receptors. *Cellular and Molecular Life Sciences*.

[18] van der Kaay D., Deal C., de Kort S. (2009). Insulin-like growth factor-binding protein-1: serum levels, promoter polymorphism, and associations with components of the metabolic syndrome in short subjects born small for gestational age. *The Journal of Clinical Endocrinology and Metabolism*.

[19] Nepstad I., Hatfield K. J., Gronningsaeter I. S. (2019). Effects of insulin and pathway inhibitors on the PI3K-Akt-mTOR phosphorylation profile in acute myeloid leukemia cells. *Signal Transduction and Targeted Therapy*.

[20] Godoy-Parejo C., Deng C., Liu W., Chen G. (2019). Insulin stimulates PI3K/AKT and cell adhesion to promote the survival of individualized human embryonic stem cells. *Stem Cells*.

[21] Molinaro A., Becattini B., Mazzoli A. (2019). Insulin-driven PI3K-AKT signaling in the hepatocyte is mediated by redundant PI3Kalpha and PI3Kbeta activities and is promoted by RAS. *Cell Metabolism*.

[22] Wang G., Shi B., Fu Y. (2019). Hypomethylated gene *NRP1* is co-expressed with *PDGFRB* and associated with poor overall survival in gastric cancer patients. *Biomedicine & Pharmacotherapy*.

[23] Huang F., Wang M., Yang T. (2014). Gastric cancer-derived MSC-secreted PDGF-DD promotes gastric cancer progression. *Journal of Cancer Research and Clinical Oncology*.

[24] Hayashi Y., Bardsley M. R., Toyomasu Y. (2015). Platelet-Derived Growth Factor Receptor-*α* Regulates Proliferation of Gastrointestinal Stromal Tumor Cells With Mutations in *KIT* by Stabilizing ETV1. *Gastroenterology*.

[25] Fukuoka S., Hara H., Takahashi N. (2020). Regorafenib plus nivolumab in patients with advanced gastric or colorectal cancer: an open-label, dose-escalation, and dose-expansion phase Ib trial (REGONIVO, EPOC1603). *Journal of Clinical Oncology*.

[26] Xu B., Bai Z., Yin J., Zhang Z. (2019). Global transcriptomic analysis identifies *SERPINE1* as a prognostic biomarker associated with epithelial-to-mesenchymal transition in gastric cancer. *PeerJ*.

[27] Liu J., Liu Z., Zhang X., Gong T., Yao D. (2019). Bioinformatic exploration of OLFML2B overexpression in gastric cancer base on multiple analyzing tools. *BMC Cancer*.

[28] Chen C., Maimaiti A., Zhang X. (2018). Knockdown of RAI14 suppresses the progression of gastric cancer. *OncoTargets and Therapy*.

[29] Zhang J., Huang J. Y., Chen Y. N. (2015). Whole genome and transcriptome sequencing of matched primary and peritoneal metastatic gastric carcinoma. *Scientific Reports*.

